# Pt-Black-Modified (Hemi)spherical AFM Sensors: *In Situ* Imaging of Light-Driven Hydrogen Peroxide Evolution

**DOI:** 10.1021/acs.analchem.3c03957

**Published:** 2024-02-14

**Authors:** Andreas Hellmann, Gregor Neusser, Sven Daboss, Mohamed M. Elnagar, Johannes Liessem, Dariusz Mitoraj, Radim Beranek, Stéphane Arbault, Christine Kranz

**Affiliations:** † Institute of Analytical and Bioanalytical Chemistry, Ulm University, Albert-Einstein-Allee 11, 89081 Ulm, Germany; ‡ Institute of Electrochemistry, Ulm University, Albert-Einstein-Allee 47, 89081 Ulm, Germany; § Univ. Bordeaux, CNRS, Bordeaux INP, UMR 5248, CBMN, F-33600 Pessac, France

## Abstract

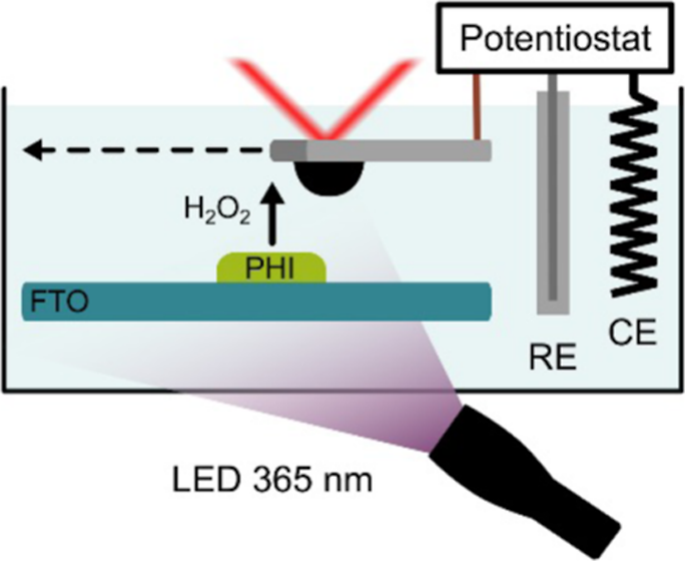

In this work, we present (hemi)spherical atomic force microscopy (AFM) sensors for the detection of hydrogen peroxide. Platinum-black (Pt-B) was electrodeposited onto conductive colloidal AFM probes or directly at recessed microelectrodes located at the end of a tipless cantilever, resulting in electrocatalytically active cantilever-based sensors that have a small geometric area but, due to the porosity of the films, exhibit a large electroactive surface area. Focused ion beam-scanning electron microscopy tomography revealed the porous 3D structure of the deposited Pt-B. Given the accurate positioning capability of AFM, these probes are suitable for local in situ sensing of hydrogen peroxide and at the same time can be used for (electrochemical) force spectroscopy measurements. Detection limits for hydrogen peroxide in the nanomolar range (LOD = 68 ± 7 nM) were obtained. Stability test and first in situ proof-of-principle experiments to achieve the electrochemical imaging of hydrogen peroxide generated at a microelectrode and at photocatalytically active structured poly(heptazine imide) films are demonstrated. Force spectroscopic data of the photocatalyst films were recorded in ambient conditions, in solution, and by applying a potential, which demonstrates the versatility of these novel Pt-B-modified spherical AFM probes.

Hydrogen peroxide (H_2_O_2_) is a highly important compound with a production volume of 4400 thousand tons in 2022.^1^ H_2_O_2_ is of significant industrial relevance, for example, serving as an oxidizing agent in production processes^2^ or as a disinfectant in the health sector.^3^ In life sciences, H_2_O_2_ has ubiquitous functions as a relative stable reactive oxygen species and as a major redox metabolite.^4–6^ To date, H_2_O_2_ is majorly produced through the energy-intensive anthraquinone process.^7^ Alternatively, an emerging field in sustainable chemistry explores green and light-driven H_2_O_2_ production from dioxygen (O_2_) and water. Among materials for photocatalytic H_2_O_2_ generation,^8–12^ polymeric carbon nitrides^13–16^ and particularly various types of poly(heptazine imide) (PHI),^17,18^ i.e., ionic variants of carbon nitride, are promising routes for light-driven H_2_O_2_ production by reducing O_2_ in the presence of oxidizing alcohols.

To evaluate the light-driven activity of the photocatalysts, the concentration of produced H_2_O_2_ is usually determined via UV–vis spectroscopy using, e.g., the titanyl oxysulfate method^17^ or by traditional redox titration using potassium permanganate.^19^ In addition, fluorescent probes^20^ are used for H_2_O_2_ determination including the commercially available Amplex UltraRed reagent, which has been applied, e.g., for the analysis of photocatalytic activity of CdS nanoparticles.^21^ However, these methods suffer from limited sensitivity and, therefore, require relatively long illumination times to achieve measurable H_2_O_2_ concentrations in bulk solutions. Local concentration changes and reaction kinetics of H_2_O_2_ generation are difficult to access, particularly for heterogeneous light-driven catalysis at microsized active materials. H_2_O_2_ can be determined electrochemically with high sensitivity using electrodes modified with electrocatalytically active layers such as Pt-black (Pt-B)^22^ or Prussian blue (PB).^23^ For example, the electrodeposition of Pt-B onto microelectrodes significantly increases the electrochemically active surface area (EASA) and shows high catalytic activity for inner sphere redox processes like H_2_O_2_ oxidation, as the high surface area provides an increased number of active sites for redox reactions.^24^ Pt-B^25^ as well as PB^23^ can be electrodeposited to obtain micronanostructured electrode surfaces under controlled growth conditions. The modification of micro- or nanoelectrodes is attractive, as the EASA is significantly increased, while the dimensions of the electrode stay in the micro- or nanometer regime. For example, Pt-B sensors have been used for measurements of intra- and extracellular H_2_O_2_ levels in living cells using modified carbon-based micro- and nanoelectrodes.^26–29^ Additionally, local H_2_O_2_ levels have been monitored at non-noble metal catalysts and at model cells via scanning electrochemical microscopy (SECM).^30,31^

Although such probes are sufficient for high-resolution reactivity maps, topographical and nanomechanical information are hardly obtained. Understanding nanomechanical properties is crucial to gain insights into many phenomena, including cell adhesion and single-molecule interactions, but also degradation processes of active materials. For operando and in situ studies, where the active material might undergo structural and morphological changes due to, e.g., degradation, such multiparametric information is of high relevance to correlate structural changes with activity. Atomic force microscopy (AFM) and derived techniques such as combined atomic force microscopy–scanning electrochemical microscopy (AFM–SECM) and colloidal probe microscopy have been successfully used for such multiparametric mappings.^32–35^ Colloidal AFM probes were introduced by Butt^36^ and Ducker et al.^37^ for force spectroscopy measurements at soft samples like biological entities or polymers, as the mechanical pressure compared to sharp AFM probes is significantly reduced. Our group introduced conductive colloidal AFM–SECM probes by utilizing metal-coated spheres instead of nonconductive silica spheres. These conductive colloidal probes are fabricated by attaching the conductive spheres to tipless, metallized, and insulated silicon-nitride cantilevers after exposing a recessed electrode via gluing the conductive colloid to the exposed area. Using this approach, AFM tip-integrated sensors were developed.^33,38,39^ A similar approach was adapted by Karg et al. using a focused ion beam (FIB)-less process to produce conductive colloidal probes.^40^ Conductive colloidal AFM probes have been used for electrochemical force spectroscopy as recently shown by Daboss et al.^39^ and Knittel et al.^38^ After modifying the conductive colloidal probes with, e.g., polymers, adhesion measurements under electrochemical conditions have been performed by applying a defined electrode potential to the colloidal probe. Daboss et al. presented electrochemical force spectroscopy measurements at the single bacterium level (*Pseudomonas fluorescens*) using a polydopamine-modified colloidal AFM–SECM probe.^39^ Although limited in achievable topographical resolution, such probes have been used for SECM imaging and conductive AFM measurements.^33^ Colloidal AFM–SECM probes may also be used to measure locally redox active molecules at low concentrations, e.g., at single entities such as micro/nanoparticles or single cells. The high surface area and geometrical dimensions of the colloid, spanning from submicrometers to a few micrometers, can be modified with sensing layers. Furthermore, given the positioning capability in AFM measurements, precise control of the distance between the sensor and the sample surface is enabled. A potential application using such colloidal AFM sensors is in situ measurements, correlating degradation associated with decay in activity with nanomechanical properties. For example, Adler et al.^41^ showed AFM studies revealing morphological changes of the alkali metal poly(heptazine imide) (K,Na–PHI) photoanodes after prolonged illumination. Hence, such structural changes and changes in nanomechanical properties may be relevant factors contributing to the material deactivation. Therefore, the correlation of mechanical/structural information with light-driven activity (H_2_O_2_ generation) via in situ methods is relevant.

Here, we present the design of AFM tip-integrated H_2_O_2_ sensors by modifying conductive colloid probes with an electroactive layer of Pt-B or by direct electrodeposition of Pt-B (denoted in the following as hemispherical Pt-B probe) onto the recessed microelectrode of the tipless cantilever, with the latter being a fast, simple, and less laborious process. In addition, we demonstrate that the modification via direct deposition is independent of the size of the attached colloids and can be tailored by the size of the recessed electrode and the deposition parameters. The hemispherical Pt-B probe was used to monitor light-driven H_2_O_2_ evolution at structured, robust, and binder-free K,Na–PHI films obtained via a sol–gel process from a water-soluble PCN precursor.^41^ The K,Na–PHI materials have previously shown high photocatalytic activity and selectivity in alcohol oxidation and H_2_O_2_ production.^42,43^ However, the potential of using Pt-B probes for mapping the local H_2_O_2_ distribution along with force spectroscopy measurements to determine the mechanical properties of such films has not been studied yet. In this respect, the developed probes can be applied in various fields, correlating mechanical properties with photocatalytic activity, the latter can be directly monitored with the onset of illumination without requiring a sampling step.

## Experimental Section

### Chemicals and Materials

Disodium hydrogen phosphate (Na_2_HPO_4_) was purchased from VWR International, USA. Potassium dihydrogen phosphate (KH_2_PO_4_), sodium chloride (NaCl), potassium chloride (KCl), hydrogen hexachloroplatinate (H_2_[PtCl_6_]), lead(II) nitrate (Pb(NO_3_)_2_), hydrogen peroxide (30% solution), ethanol absolute, and sulfuric acid (85% v/v) were obtained from Merck, Germany. Hexaammineruthenium(III) chloride ([Ru(NH_3_)_6_]Cl_3_) was purchased from Acros Organics, Belgium. Epoxy resin was obtained from Araldite, Huntsman Advanced Materials GmbH, Switzerland. All solutions were prepared with ultrapure water (Elga water system, UK, conductivity 18.0 MΩ·cm). Polishing materials were obtained from Buehler ITW Test & Measurement GmbH, Germany, and Allied High-Tech Products Inc., USA, respectively. Glass capillaries were obtained from Hilgenberg, Germany, and platinum microwires were purchased from Goodfellow, UK.

### Fabrication and Electrochemical Characterization of Pt-B-Modified Probes

Microelectrodes were prepared as described elsewhere^44^ by sealing a platinum wire (25 μm in diameter) under vacuum in borosilicate glass followed by consecutive grinding and polishing steps and finally cleaning in an ultrasonic bath for 15 min in ultrapure water. Electrochemical deposition of Pt-B was carried out according to a procedure adapted from Ben-Amor et al.^45^ by applying a constant potential of −0.06 V vs Ag/AgCl/KCl (sat.) for 40 s in a solution of 31 mM H_2_PtCl_6_ and 0.67 mM Pb(NO_3_)_2_ in phosphate-buffered saline (PBS) (pH = 3.2). A platinum counter electrode (Pt-CE) (1 mm diameter Pt wire) was used if not otherwise stated.

AFM–SECM probes were fabricated as previously described.^38^ In brief, commercially available tipless AFM cantilevers (Bruker NP-O, nominal force constant (*k*) = 0.06 N m^–1^) were modified with a gold layer via evaporation and coated with silicon oxide/silicon nitride layers via PE-CVD. Selective removal of the insulation layer was achieved through XeF_2_-assisted (insulator-enhanced etch, IEE) FIB milling (Helios Nanolab 600 or Quanta 3D FEG, Thermo Fisher Scientific, USA) to remove locally the insulation layer and expose a recessed, disk-shaped (sub)-microelectrode at the end of the cantilever. For colloidal probes, microsized (5 μm in diameter) gold-coated melamine resin beads (Microparticles, Germany) were attached using a micromanipulator in combination with an inverted microscope using Norland Optical Adhesive 81 (NOA 81, Norland Products, USA). Deposition of Pt-B directly at the recessed microelectrode or at the attached colloidal probe was achieved in a three-electrode setup using a Pt-CE, by applying a constant potential of −0.06 V vs Ag/AgCl/KCl (sat.) for 10 s in a solution containing 31 mM H_2_PtCl_6_ and 0.67 mM Pb(NO_3_)_2_ in PBS.

The individual modification steps were monitored by cyclic voltammetry in a three-electrode setup using a CHI 660C potentiostat (CH Instruments, USA) in a 10 mM [Ru(NH_3_)_6_]Cl_3_/0.1 M KCl solution at a scan rate of 0.1 V s^–1^. To determine the EASA, the potential was cycled between 0.4 and 1.4 V at a scan rate of 0.2 V s^–1^ in 0.5 M H_2_SO_4_ solution vs a Hg/Hg_2_SO_4_ reference electrode until a stable voltammogram was obtained. H_2_O_2_ calibrations were performed in PBS solution at pH = 7.4 by using chronoamperometry. The Pt-B-modified probes were biased at 0.35 V vs Ag/AgCl/KCl (sat.) following consecutive additions of H_2_O_2_ aliquots resulting in a concentration of 2.5 μM per addition.

### Focused Ion Beam-Scanning Electron Microscopy (FIB-SEM) Characterization

To minimize beam-induced damage during FIB-SEM analysis, we used the micromanipulator in combination with an inverted microscope to embed the attached colloid in a protective epoxy resin layer following a similar procedure published by T. Philipp et al.^46^ To ensure that the porous structure of Pt-B is infiltrated with epoxy, two vacuum steps (240 mbar, 5 and 30 min, respectively) were applied. After embedding, the AFM probe was coated with approximately 10 nm of platinum using sputter coating (SCD 005, BAL-TEC, Liechtenstein). For FIB-SEM studies, an additional layer of platinum was deposited onto the embedded electrode using ion beam-induced deposition with methylcyclopentadienyl trimethyl platinum (C_9_H_16_Pt) as a precursor. To generate the tomography data sets and to avoid redeposition during FIB milling, material left and right of the Pt-B electrode was removed by FIB milling, leaving a rectangular area of 6 μm × 6 μm. Ultimately, the front facet of the block was cleaned via a cleaning cross section at 30 kV and 90 pA. Sectioning was performed using software module Auto Slice & View.G1 (Thermo Fisher Scientific, USA). With each step, a slice with a thickness of 30 nm of material was removed by FIB, and the new block face was imaged with the electron beam operated at an accelerating voltage of 3 kV and a beam current of 86 pA using the through-the-lens detector. The size of one voxel was 7.14 × 7.14 × 30 nm^3^.

### Data Processing

The obtained electron image stacks of the Pt-B probes were all processed with an Avizo Lite 2020.2 (Thermo Fisher Scientific, USA) and the open-source software package Fiji.^47^ The image stacks were aligned using the gold layer and transition to the side wall as reference points on all recorded images. Besides bleach correction^48^ and median filter, image stacks of the Au colloidal Pt-B probe were additionally processed with a lateral brightness correction using the plane brightness adjustment plugin in Fiji.^49^ Finally, the image stacks were binarized, segmented, and visualized in 3D.

### AFM and AFM–SECM Measurements

All AFM measurements were performed with a 5500 AFM/SPM microscope (Keysight Technologies, USA) in contact mode either in air or in solution. Imaging of the H_2_O_2_ generating Pt-ME (bias: −0.6 V vs Ag/AgCl, WE2) was done after retracting the hemispherical Pt-B-modified probe (biased at 0.35 V vs Ag/AgCl, WE1) by a distance of 20 μm (false engagement). A Pt wire was used as a counter electrode. Images were recorded with a scan rate of 0.2 ln s^–1^ in PBS at pH = 7.4. For H_2_O_2_ measurements at the structured photocatalyst sample, 20% ethanol was added to the solution, and an optical fiber (diameter 1000 μm, M59-L01, Thorlabs GmbH, Bergkirchen, Germany) connected to a 141 mW blue LED (365 nm, M365FP1, Thorlabs GmbH) was used to illuminate the K,Na–PHI photoelectrode. For control measurements, commercially available silicon nitride probes (ORC-8, Bruker AFM probes, USA) were used. The mean surface roughness was determined in three different areas of the sample surface.

### Force Spectroscopy

The force constant of the AFM cantilever was determined using the thermal noise method.^50^ Force distance curves at K,Na–PHI photocatalysts and fluorine-doped tin oxide (FTO) substrate were recorded in air and PBS solution (with and without applied potential at the Pt-B probe) at a sweep rate of 1.0 μm s^–1^ with loading forces of 200 nN. In total, force curves were recorded at three individual spots (50 force curves in air or 60 force curves in solution and under applied potential), giving a total number of 150 (180) data points, respectively. The measured adhesion forces are reported as the mean standard deviation.

### Preparation of the K,Na–PHI Photocatalyst

The synthesis of the photocatalyst and coating of the FTO substrate followed the procedures published by Krivtsov et al.^42^ and Adler et al.^41^ For further details, the reader is referred to the Supporting Information.

### COMSOL Simulation

The electrochemistry module of COMSOL Multiphysics (Version 6.0) was used to model the diffusion profiles toward the recessed and colloidal AFM–SECM probe. The used geometry is displayed in Figure S1 showing the key boundaries. Boundary dimensions such as the electrode surface and nonconductive parts were set according to the experimentally used probes. A mesh was then formed at the surface, and a refined denser mesh was used to describe the active electrode surface and surrounding areas where variations of local flux and local concentration are expected. Based on the Butler–Volmer equation and Faraday’s laws, the electrode surface boundary conditions are defined and are assigned to each mesh element. The total current at the electrode was then determined by integrating the local current density across the electrode surface. The resulting concentration profiles are shown in Figure S2 and the response in the presence of the sample in Figure S10, respectively.

## Results and Discussion

### Fabrication and SEM-FIB Characterization

**Figure 1:**
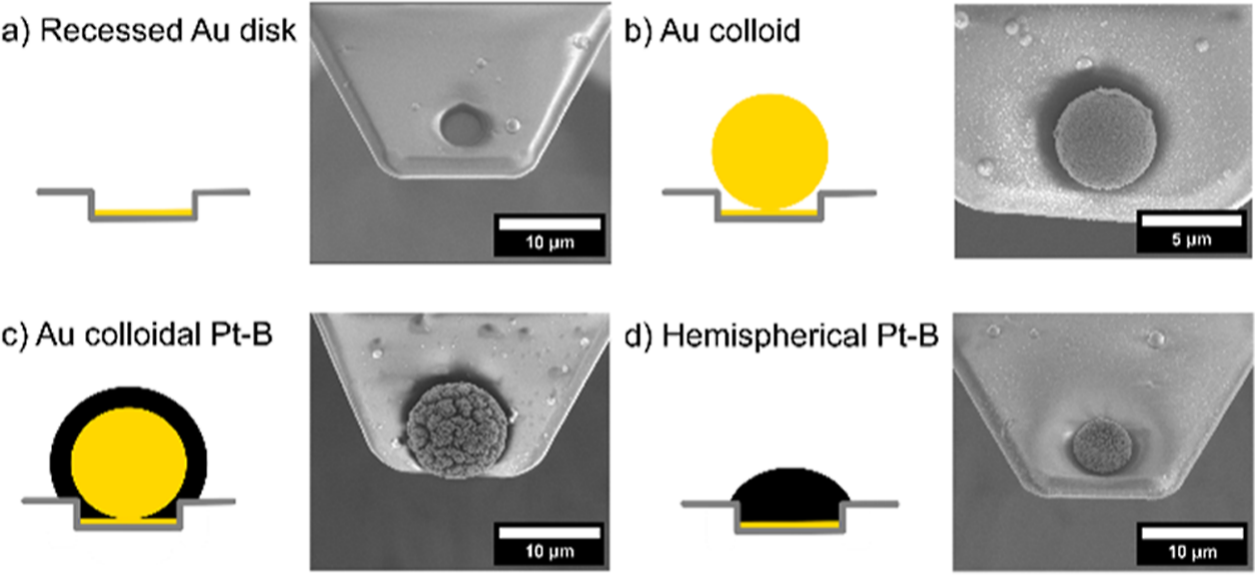
SEM images of (a) recessed Au disk microelectrode, (b) after Au colloid attachment, (c) after electrodeposition of Pt-B on the Au colloid, and (d) after electrodeposition of Pt-B directly on the recessed Au disk electrode. Electrodeposition of Pt-B was carried out in 31 mM H_2_PtCl_6_/0.67 mM Pb(NO_3_)_2_ in PBS by applying a constant potential of −0.06 V vs Ag/AgCl for 10 s.

**Figure 2:**
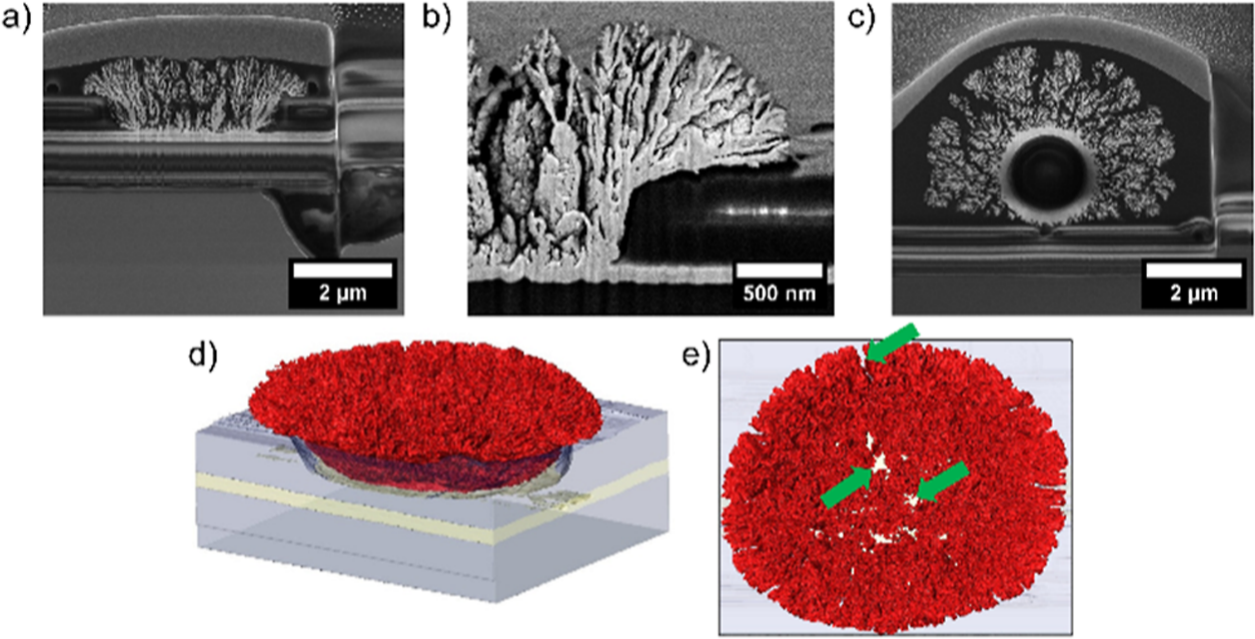
Secondary electron (SE) image of the cross section of the (a) hemispherical Pt-B probe and (b) backscattered electron image of the cross section. SE image of the cross section (c) of the Au colloidal Pt-B probe. 3D reconstruction of the (d) recessed microelectrode modified with the Pt-B film from the side and (e) from top, respectively (for the attached, modified colloids, no tomography sets were recorded). For better visualization, false color images are used: the surface of Pt-B is marked in red, the gold layer is displayed in yellow, the Si_*x*_N_*y*_/SiO_2_ insulation layer is marked in gray, and channels within the porous material are marked by green arrows.

We present here two strategies for the fabrication of Pt-B-modified colloidal AFM–SECM probes starting from a milled recessed Au electrode (Figure 1a). Based on previous results,^38^ a commercially available Au colloid is attached to a modified tipless cantilever bearing a recessed gold disk microelectrode (diameter 4 μm), as shown in Figure 1b, which was subsequently modified via electrodeposition of Pt-B (Figure 1c). Alternatively, Pt-B can be directly electrodeposited onto the recessed Au electrode (Figure 1d). SEM micrographs (Figure 1c,d) of the Pt-B-modified probe exhibit a cauliflower-like structure, which is in accordance with the observations of Badets et al.^24^ The directly deposited Pt-B forms a truncated half-sphere, as shown in Figure 1d. Given the reduced number of fabrication steps, we will mainly focus on the discussion of the hemispherical Pt-B probe. We used FIB cross sections to reveal the internal structure of the Pt-B deposits (Figure 2a–c) and performed FIB-SEM tomography to obtain the reconstructed 3D images (Figure 2d,e). Based on previous findings on the electrodeposition of Pt-B on microelectrodes,^51^ we chose a deposition time of 10 s at −0.06 V vs Ag/AgCl for the recessed Au disk electrode to prevent uncontrolled overgrowth of the film. Consequently, the diameter of the initial recessed Au microelectrode increases from 4.0 to 5.1 μm for the direct deposition of Pt-B. The thickness was estimated to be 1.9 ± 0.1 μm (*n* = 5) from the SEM image of the cross section (Figure 2a). The resulting electrode has a hemispherical shape and a height of about 1.0 μm. The top view of the 3D reconstruction (Figure 2e) reveals a porous material with channels and gorges that pervade throughout the Pt-B volume (indicated by green arrows). Using Avizo software, a total surface area of 606 μm^2^ for the hemispherical Pt-B electrode was determined from the FIB-SEM tomography data set. It should be noted that the determination of the surface area is limited by the voxel size (7.14 × 7.14 × 30 nm^3^). We also fabricated smaller hemispherical probes with Pt deposits of approximately 700 nm in diameter (see Figure S3a and inset) by depositing Pt directly onto recessed Au disk electrodes with a diameter of 500 nm. These smaller probes were fabricated using a pulsed deposition approach in the absence of Pb(NO_3_)_2_. As a proof of principle, we also fabricated other noble metal probes such as palladium (Pd) hemispherical probes, which may be used in future studies for local hydrogen measurements (Figure S3c). We found under similar conditions the formation of Pd colloids with a size of approximately 2.5 μm deposited from K_2_PdCl_6_ solution onto recessed Au disk electrodes (diameter 1 μm) (Figure S3c and inset) using again a pulse deposition technique.^52^ The respective cyclic voltammograms of these smaller probes are shown in Figure S3b,d, respectively. Pd-modified microelectrodes are suitable for in situ mapping of H_2_ as demonstrated in a recent contribution.^53^ An advantage of this direct deposition process is that the size can be tailored by the deposition parameters, and the fabrication of submicrometer-sized sensors is possible, as it is not limited by the size of the mechanically attached colloids. Hence, in the following, we focused on the Pt-B hemispherical probe.

### Electrochemical Characterization and Analytical Figures of Merit

**Figure 3:**
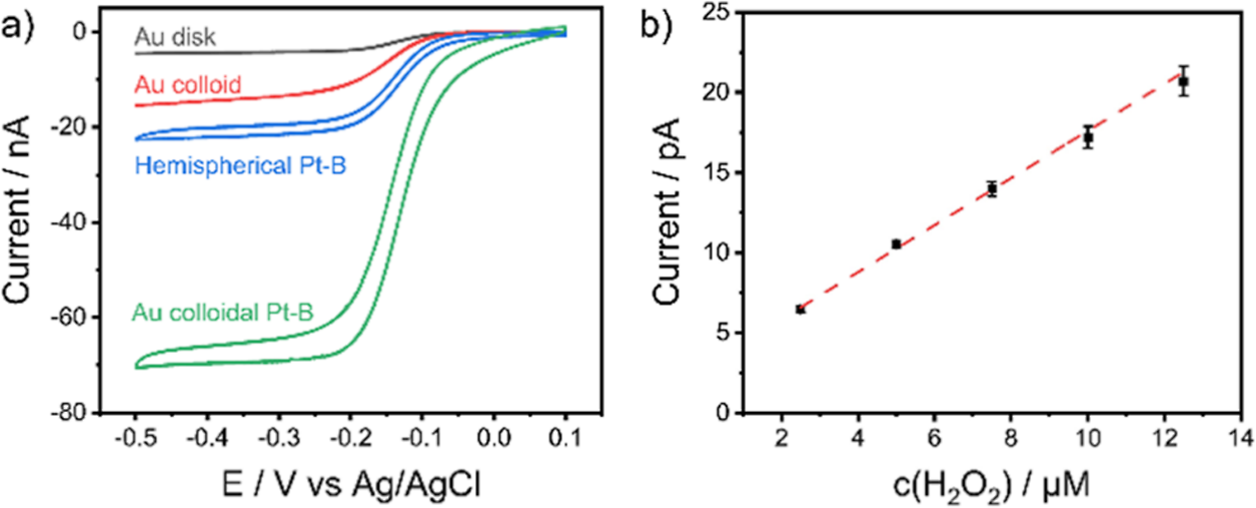
a) CVs recorded in 10 mM [Ru(NH_3_)_6_]Cl_3_/0.1 M KCl vs Ag/AgCl of the individual processing steps, scan rate: 0.1 V s^–1^, and (b) calibration curve of the hemispherical Pt-B probe for H_2_O_2_ in PBS at pH 7.4 (*E*_probe_ = 0.35 V vs Ag/AgCl, error bars reflect three measurements recorded with the same probe).

The individual modification steps of the cantilever were characterized via cyclic voltammetry in a [Ru(NH_3_)_6_]Cl_3_/0.1 M KCl solution to determine the steady-state currents (*i*_ss_) (Figure 3a).

The experimentally obtained cyclic voltammograms and steady-state currents (*i*_ss_) (Figure 3a) for the recessed Au disk microelectrode and Au colloid AFM–SECM probe are in excellent agreement with the theoretically obtained current values (Figure S4). As expected and shown in Figure 3a, the steady-state current, *i*_ss_, increases in the sequence of Au colloidal Pt-B > hemispherical Pt-B > Au colloid > Au disk. While for the Au colloidal Pt-B probe, an increase of *i*_ss_ by Δ*i*_ss_ = 54 nA (365%) was observed in comparison to the nonmodified Au colloidal microelectrode, for the hemispherical Pt-B probe, only an increase of Δ*i*_ss_ = 18 nA (390%) was obtained. However, given the reduced number of fabrication steps, this fabrication approach is less laborious, and direct electrodeposition still increases *i*_ss_ by approximately 390%, still leading to a high electroactive surface area. To calculate the EASA of the hemispherical Pt-B probe, the hydrogen desorption peak detected in H_2_SO_4_ at approximately −0.2 V was integrated (Figure S5).^54^ The obtained integral was divided by the scan rate, which corresponds to a total EASA of 5183 ± 235 μm^2^ (*n* = 3), assuming the formation of a monolayer with the respective charge of *Q* = 208 μC cm^–2^. The calculated EASA is 9-fold larger compared to the surface area determined via FIB-SEM tomography. Notably, a linear calibration (*R*^2^ = 0.9964) with good reproducibility of repeated measurements is achieved for H_2_O_2_ using the hemispherical Pt-B-modified probe in PBS solution, as illustrated in Figures 3b and S6, respectively. Furthermore, the developed probe possesses an excellent sensitivity of 1.47 μA M^–1^, as calculated from the initial slope of the calibration curve. The sensor-to-sensor variation was also determined for the hemispherical Pt-B probes and yielded a variation coefficient of 7% (σ/m) among the three individual probes. The limit of detection (LOD), calculated from the slope of the calibration function (Figure 3b) using three times the standard deviation, resulted in an LOD of 68 ± 7 nM for three consecutive measurements.

### In Situ Topography and H_2_O_2_ Mapping

**Figure 4:**
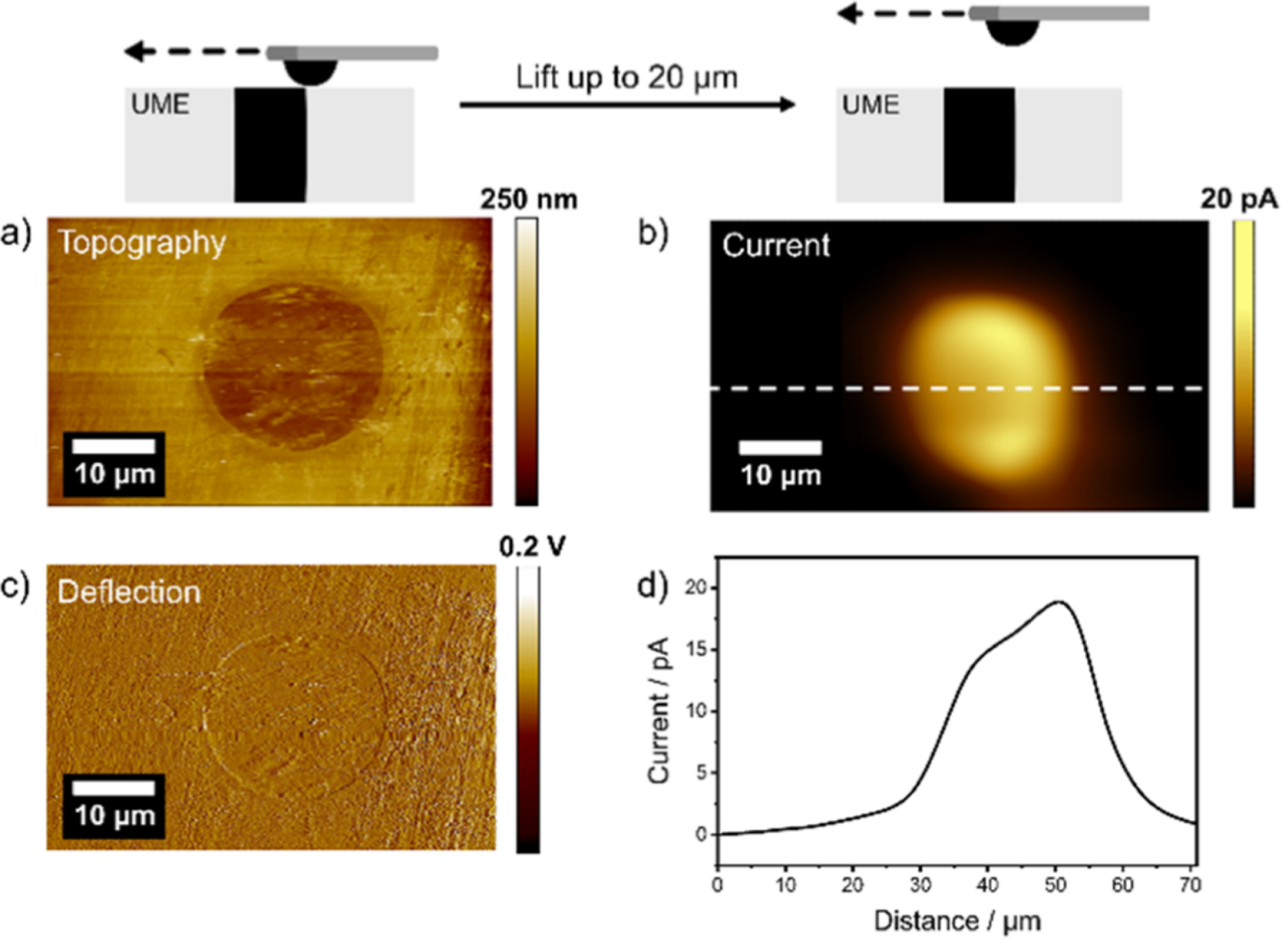
a) Contact mode AFM topography image. (b) False engagement current image of the H_2_O_2_ evolution at the Pt-ME (bias: −0.6 V vs Ag/AgCl, WE2) after the hemispherical Pt-B probe (biased at 0.35 V vs Ag/AgCl, WE1) was retracted to 20 μm and (c) deflection image of a Pt-ME (25 μm in diam.) recorded with the hemispherical Pt-B probe in PBS at pH = 7.4. (d) Respective current profile indicated as a dashed white line in (b). All images were recorded with a scan speed of 0.2 ln s^–1^.

Mapping of H_2_O_2_ generation via SECM using, e.g., Pt-B- or PB-modified microelectrodes,^55^ is an attractive approach to study local catalytic activity and derive mechanistic insights into oxygen reduction reaction. The generation of electroactive species such as hydrogen or H_2_O_2_ at microelectrodes has been mapped via SECM.^53,55,56^ As a first step, we investigated the mechanical stability of the hemispherical Pt-B probe by scanning a Au substrate (area: 30 × 30 μm^2^) in contact mode (data not shown). The SEM images (Figure S7) before and after the scan clearly reveal the high mechanical stability of the hemispherical Pt-B probe; no obvious changes in the porous electrode structure were observed. In a first proof-of-principle experiment, the hemispherical Pt-B probe was used to map the topography of a Pt-disk microelectrode (diameter 25 μm) using contact mode AFM in PBS solution. As shown in Figure 4a,c, the imaging quality is very good, showing detailed features (see deflection image) which are much smaller than the Pt-B hemisphere. We assume that due to the roughness of the Pt deposit, a spiky feature of the Pt-B film acts as the AFM tip, providing the high-resolution image of the flat model sample. After retracting the probe by 20 μm, potentials were simultaneously applied to the AFM probe (W1: 0.35 V vs Ag/AgCl) and the Pt microelectrode (W2: −0.6 V vs Ag/AgCl). The evolution of H_2_O_2_ at the microelectrode was mapped in SECM generation/collection mode^57^ in constant height (“false engagement”) to avoid short circuiting between the two electrodes. As expected, the recorded faradaic current at the AFM probe (SECM image) corresponds to the size and shape of the sealed Pt microdisk (Figure 4b). It should be noted that, as true for all SECM measurements in the constant height mode, the current image shows an averaged diffusion profile whose resolution, also in terms of local heterogeneity, depends on the substrate–probe distance. COMSOL simulations (Figure S10) were carried out using the Pt-B hemisphere in a fixed position, while the UME was moved in respect to the probe position in a fixed plan. The current profile is shown in Figure S10a, while the respective line (x)-scan (Figure S10b) shows a peak with a maximum current of ca. 20 pA fitting very well the SECM experiment, and the current profile as shown in Figure 4d. A significant broadening of the SECM response was not observed, which we attribute to the shape of the Pt-B probe and the fact that the majority of the signal comes from a small area directly facing the sample.

For heterogeneous photocatalysts, standard methods like UV–vis spectroscopy or head space gas chromatography are used to determine the activity rate face challenges of bulk measurements including sampling steps and limitation in online monitoring of photocatalytic activity.^58^ SECM has been proven to be an attractive electroanalytical method to map and quantify light-driven and photoelectrocatalytic activity at heterogeneous systems.^59–63^ Here, we investigate the generation of H_2_O_2_ at K,Na–PHI films which are attractive because they exhibit photocatalytic activity without the need for applying an external potential in the presence of sacrificial electron donors, such as ethanol or benzyl alcohol.^17,42^ To demonstrate the generation of H_2_O_2_ in the presence of ethanol, Pt-B-modified microelectrodes (diameter 25 μm) were used to determine the H_2_O_2_ amount at the pristine (i.e., unstructured) K,Na–PHI photocatalytic film. The microelectrode was positioned at a distance of 50 μm to the K,Na–PHI surface by recording the SECM approach curve (current–distance curve) using the oxygen reduction current (microelectrode biased at −0.6 V vs Ag/AgCl) under dark conditions. Then, a potential of 0.35 V vs Ag/AgCl was applied in a PBS solution containing 20% ethanol. Illumination was carried out using a LED (λ = 365 nm), which resulted in an immediate current response of approximately 200 pA due to the oxidation of H_2_O_2_ at the microelectrode, which is formed upon the light-driven reduction of O_2_ in the presence of ethanol, as shown in Figure S8a. After initial generation of H_2_O_2_, the signal decreases slightly and reaches a steady-state value. This may be related to the fact that an equilibrium is reached within the solution. The same behavior is observed upon continuously switching on and off the light source (chopped mode) (Figure S8b). Based on Faraday’s law, we determined the rate of H_2_O_2_ generation from the last three illumination steps to be 25.1 ± 0.7 nM s^–1^ which is in accordance with UV–vis spectroscopic measurements considering the longer illumination times (up to 4 h).^42^ It should be noted that the results cannot be compared directly as the experimental parameters (e.g., used sacrificial electron donor, pH value, etc.) were different between the homogeneous^42^ and heterogeneous photocatalytic experiments. As a control experiment, catalase was added to solution during the illumination, which led to a decrease in the current signal (Figure S8c) due to the catalytic conversion of the produced H_2_O_2_ into O_2_ and H_2_O. The decrease in current given the high selectivity of catalase for the conversion of H_2_O_2_ clearly reveals that H_2_O_2_ is predominantly produced in a two-electron step rather than H_2_O in a four-electron pathway. Through the photoexcitation of the K,Na–PHI catalyst in the presence of alcohol as a sacrificial electron donor, a hydroperoxide intermediate is formed. The intermediate species immediately decomposes, resulting in H_2_O_2_.^18^

**Figure 5:**
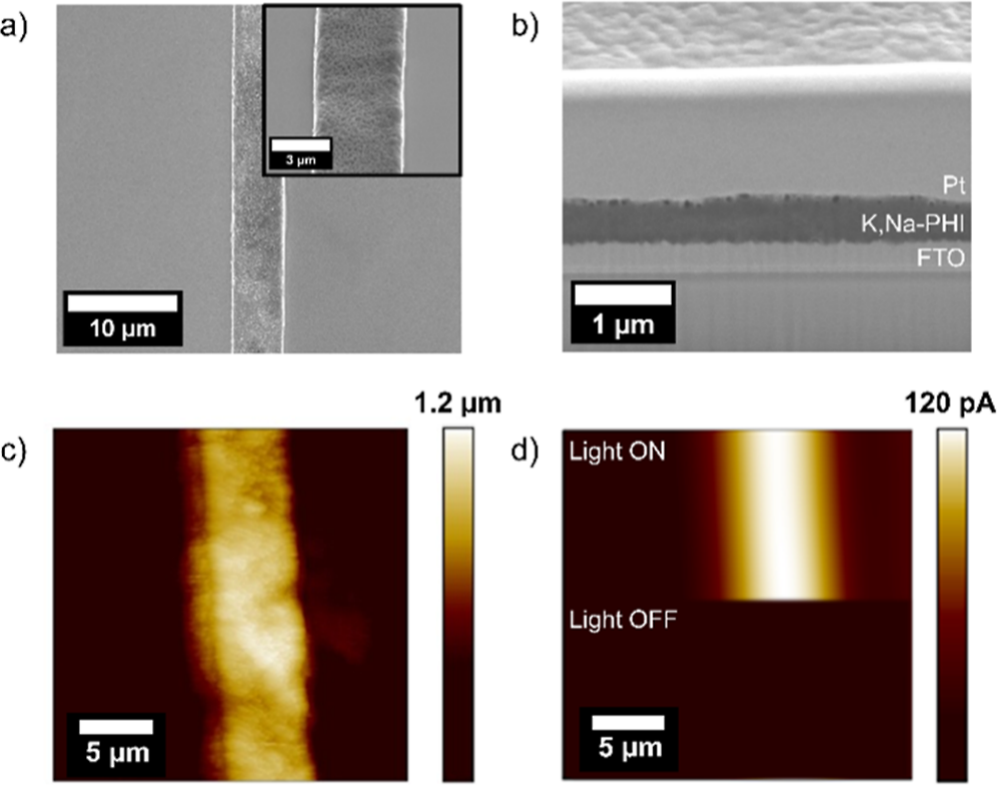
a) SEM image of the K,Na–PHI photocatalyst with the exposed 5.0 μm ridge via FIB milling; inset: zoomed view. (b) FIB cross section of the K,Na–PHI-coated FTO substrate. (c) AFM topography of the milled K,Na–PHI ridge acquired with the hemispherical Pt-B probe. (d) False engagement current image of the respective PHI ridge under illumination at λ = 365 nm. The hemispherical Pt-B probe was retracted to a distance of 20 μm and biased at 0.35 V vs Ag/AgCl in PBS and 20% EtOH. All AFM images were recorded with a scan speed of 0.2 ln s^–1^.

To demonstrate that the Pt-B-modified AFM–SECM probes are suitable for imaging, we used FIB milling to expose a 5.0 μm ridge of the K,Na–PHI photocatalytic film (Figure 5a and inset).

The K,Na–PHI layer is clearly visible in the SEM image of the FIB cross section of the pristine substrate (Figure 5b). Importantly, it exhibits no signs of detachment from the surface, which has been a problematic issue observed in PCN-based films.^41^ The hemispherical Pt-B probe was used to map the topography of the photocatalyst in the contact mode, as shown in Figure 5c prior to applying a potential to the AFM probe. As clearly visible, the AFM topography image is characterized by artifacts, as this sample has a ridge and more morphological features compared with the Pt-disk microelectrode (Figure 4). We suspect that multiple spiky features of the Pt-B probe act as a tip while scanning across the ridge, thus leading to topographical images showing artifacts. However, despite limitations in imaging quality (horizontal resolution), the height of the K,Na–PHI microstructure recorded with the hemispherical Pt-B probe fits well with the height determined with a commercial AFM probe (nominal tip radius of 15 nm) (Figure S9) and FIB-SEM cross sections (Figure 5b). After retracting the hemispherical Pt-B probe by 20 μm, the K,Na–PHI microstructure was imaged again now under illumination to map the H_2_O_2_ evolution. A current increase of 120 pA was observed, while the probe was scanned under the illuminated K,Na–PHI ridge (Figure 5d). The current response fits well with experiments obtained with the Pt-B-modified microelectrodes recorded at the same sample before structuring. A generation rate of 3.1 fmol/s of H_2_O_2_ was determined for one single line scan with the hemispherical Pt-B probe. In contrast, no current increase was observed under dark conditions, as clearly visible when the light was switched off. To demonstrate interprobe reproducibility, the variation in the surface roughness of K,Na–PHI was determined using three individual probes, yielding a variation coefficient of only 2.6% (σ/m) (Figure S11a). In addition, the trace and retrace images are shown in Figure S11b,c, respectively. The topography of a single K,Na–PHI particle (Figure S12a–c) was imaged using a commercial AFM probe, the described hemispherical Pt-B probe with 5.1 μm diameter, and the Pt probe with 0.7 nm diameter shown in Figure S3a. The Pt probe with a small diameter gave a similar resolution compared to the commercial AFM tip and hence quite good imaging quality.

Possible contaminations originating from the hemispherical Pt-black probes during contact mode AFM imaging were not observed in the energy dispersive X-ray spectroscopy measurements of the samples after imaging. The repetitive measurements (*n* = 3) of the Pt-microelectrode (Figure 4) did not result in increased current values due to catalytically active Pt nanoparticles originating from the hemispherical probe.

### Force Spectroscopy

**Figure 6:**
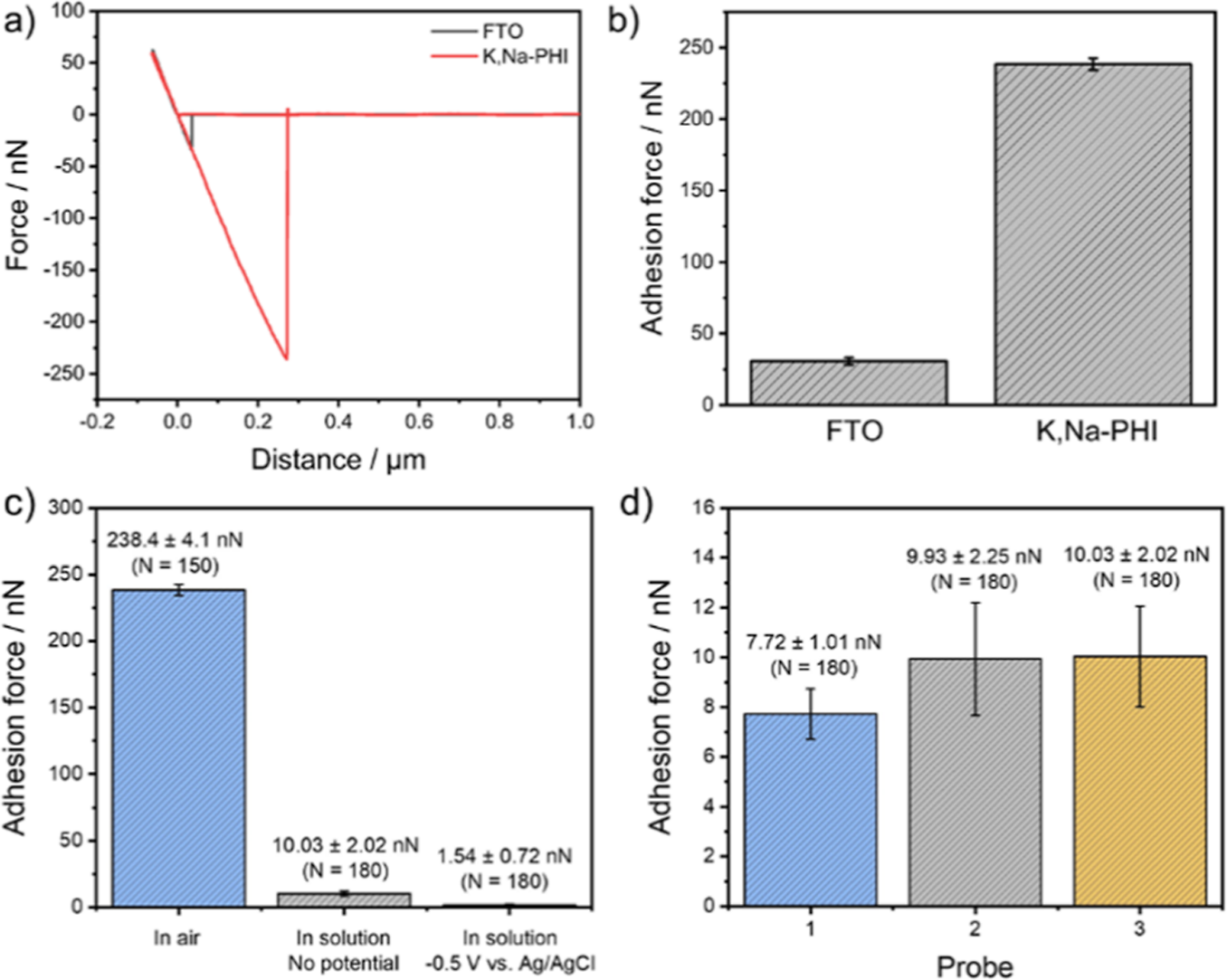
a) Exemplary force curves (approach and retract curves) on FTO and K,Na–PHI recorded with the hemispherical Pt-B probe in air. (b) Bar chart of the measured adhesion forces (error bars reflect 150 measurements of the same sample). (c) Bar chart of adhesion force at K,Na–PHI in air, PBS solution, and in PBS solution with the Pt-B probe biased at −0.5 V vs Ag/AgCl and (d) bar chart of adhesion force for three individual probes on K,Na–PHI in PBS solution.

Conductive colloidal probes for electrochemical force spectroscopy have been introduced by Knittel et al.^38^ Based on the Hertz theory of contact,^64^ quantitative physical properties such as adhesion and Younǵs modulus can be derived via the Derjaguin–Muller–Toporov model.^65^ As previously shown, colloidal probes are beneficial to determine the nanomechanical properties of soft samples as the increased contact area significantly decreases the excreted mechanical pressure,^66,67^ which was also demonstrated for conductive colloidal probes.^33,38,39^ As a proof-of-principle measurement, we performed force spectroscopy measurements with the hemispherical Pt-B-modified probe at the K,Na–PHI film and the FTO substrate (Figure 6).

As expected, the adhesion force recorded with the hemispherical Pt-B probe at K,Na–PHI is significantly larger (238.4 ± 4.1 nN, *n* = 150) compared to the FTO substrate (30.8 ± 2.6 nN, *n* = 150). Electrostatic interactions such as induced dipoles are expected between the sample surface and the tip, which is initially neutral or slightly negatively charged due to partial oxidation of Pt, with Pt–O species present at the surface, leading to stronger adhesion. Furthermore, functional groups on the surface of the K,Na–PHI photocatalyst such as –NH, –NH_2_ groups, uncondensed NH_*x*_-containing triazine species, and oxygenated (e.g., cyamelurate) surface moieties may interact with the Pt-B-modified probe, e.g., through hydrogen bonding between NH and Pt–O species. In contrast to FTO, where mainly oxide groups are present, electrostatic repulsion is expected, which fits well with our data, as the adhesion force for FTO is significantly lower compared to the K,Na–PHI film (Figure 6b). In addition, we expect increased interaction in the case of K,Na–PHI due to increased surface roughness. A mean roughness (*S*_a_) of the K,Na–PHI of 45.9 ± 3.5 nm (*n* = 3) was determined with an overall increased roughness in comparison to that of the FTO surface (*S*_a_ 18.7 ± 2.7 nm, *n* = 3). The adhesion force was also investigated in the PBS solution (Figure 6c). As expected, significantly lower adhesion forces of 10.03 ± 2.02 nN (*n* = 180) were observed due to, e.g., the absence of capillary forces. If the probe is biased at a negative potential (−0.5 V vs Ag/AgCl), adhesion forces are further reduced, which is in accordance with the expected negative charge of the K,Na–PHI film;^42^ thus, increased repulsive forces are predominant and lead to lower adhesion (1.54 ± 0.72 nN, *n* = 180). If the probe is biased at a positive potential (+0.35 V vs Ag/AgCl), then no significant change in adhesion was observed in comparison to the unbiased probe (data not shown). In addition, the interprobe reproducibility was determined in solution showing only minimal variation between individual probes (Figure 6d).

## Conclusions

In this work, we demonstrate fabrication strategies for (hemispherical)spherical electrochemical AFM-tip-integrated sensors. As a simple way to fabricate such sensors, direct deposition of Pt-B onto the recessed electrode at the tipless cantilever results in hemispherical Pt-B sensors with tunable sizes and avoids the additional attachment of a colloid, which is the established fabrication scheme. These probes were used for in situ mapping of light-driven H_2_O_2_ at structured photocatalytic K,Na–PHI films. Given the large surface area determined via FIB-SEM tomography and the significantly increased EASA determined by cyclic voltammetry, the probes combine high sensitivity for H_2_O_2_ detection with the advantage that such probes can be precisely positioned by using the electronic feedback system of the AFM instrument. In addition, force spectroscopy measurements can be recorded without changing the probe. For example, the adhesion properties of K,Na–PHI using such hemispherical Pt-B probes were investigated. The developed approach may also be of substantial interest for studies of other complex samples where control of the exerted force and the mapping of nanomechanical properties along with recording of electroactive species generated at the sample is of interest.

## Supporting Information Available

COMSOL-based simulations of diffusion mass transport of [Ru(NH_3_)_6_]Cl_3_ redox species at flat and colloidal probes; characterization of Pt-B- and Pd-modified probes by SEM and voltammetry with [Ru(NH_3_)_6_]Cl_3_ and H_2_SO_4_; amperometry study for the sensitive detection of H_2_O_2_ by Pt-B probes; SEM of a Pt-B probe before and after AFM imaging of a gold surface; amperometric detection by a Pt-B probe of H_2_O_2_ generated following a Na,K–PHI film photoillumination; AFM topography of the film ridge; COMSOL simulation of the H_2_O_2_ profile at the microelectrode and simulated line scan; surface roughness of the K,Na–PHI film determined with different Pt-B probes, and AFM topography of a single K,Na–PHI particle obtained with different (Pt-B) AFM probes (PDF)
