# Supplementary material for: Pt-Black-Modified (Hemi)spherical AFM Sensors: In Situ Imaging of Light-Driven Hydrogen Peroxide Evolution
Source: Anal Chem. 2024 Feb 14;96(8):3308–17. doi: 10.1021/acs.analchem.3c03957 (PMC10902814; doi:10.1021/acs.analchem.3c03957)
Supplement: Supplementary file 1 — ac3c03957_si_001.pdf [file ac3c03957_si_001.pdf]

Supporting Information

Pt-black-modified (hemi)spherical AFM sensors: *in situ* imaging of light-driven hydrogen peroxide evolution

Andreas Hellmann[a], Gregor Neusser[a], Sven Daboss[a], Mohamed M. Elnagar[b], Johannes Liessem[b], Dariusz Mitoraj[b], Radim Beranek[b], Stéphane Arbault[c], Christine Kranz[a]*

[a]Institute of Analytical and Bioanalytical Chemistry, Ulm University, Albert-Einstein-Allee 11, 89081 Ulm, Germany

[b]Institute of Electrochemistry, Ulm University, Albert-Einstein-Allee 47, 89081, Ulm, Germany

[c]Univ. Bordeaux, CNRS, Bordeaux INP, UMR 5248, CBMN, F-33600 Pessac, France

*E-mail: christine.kranz@uni-ulm.de

Table of Contents

Preparation of the K,Na-PHI photocatalyst

Figure S1. Boundary mesh for COMSOL simulation of diffusion.

Figure S2. Simulated concentration profile based on diffusion-controlled mass transport of $[Ru(NH_3)_6]Cl_3$.

Figure S3. SEM images and respective CVs of hemispherical Pt-B and Pd probes.

Figure S4. Simulated linear sweep voltammograms for the reduction of $[Ru(NH_3)_6]Cl_3$.

Figure S5. Cyclic voltammogram of a hemispherical Pt-B probe in sulfuric acid.

Figure S6. Amperometric *i*-*t*-curve of an exemplary H_2O_2 calibration curve recorded at a hemispherical Pt-B modified probe.

Figure S7. SEM images of the hemispherical Pt-B probe before and after imaging an Au substrate in contact mode.

Figure S8. Amperometric (*i*-*t*) curves recorded at Pt-B modified microelectrode during illumination of the K,Na-PHI film with and without adding catalase.

Figure S9. AFM topography of the K,Na-PHI ridge recorded with a commercial AFM probe.

Figure S10. COMSOL simulation of the H_2O_2 profile at the microelectrode and the simulated line scan.

Figure S11. Surface roughness of the K,Na-PHI film determined with different Pt-B probes.

Figure S12. AFM topography of K,Na-PHI particle obtained with different AFM probes.

Figure S13. Histograms of the adhesion forces recorded at K,Na-PHI and at FTO.

EXPERIMENTAL SECTION

Supplemental methods

Preparation of the K,Na-PHI photocatalyst solution. According to the procedure of Krivtsov et al.,[1] 10 mmol KOH (56 g mol^{-1}) and 5 mmol NaOH (40 g mol^{-1}) were ground and mixed with 12 mmol melamine (126 g mol^{-1}). The powder was then heated in a lid-covered crucible at a rate of 5.0 °C min^{-1} up to 330 °C, then it was kept for 2h at the reached temperature before being removed from the muffle furnace. The obtained orange solid was suspended in 100 mL of deionized water and the insoluble part was filtered out, firstly, by a paper filter and afterward by a 0.2 μm PTFE syringe filter. The filtered solution was filled inside a cellulose membrane sack with a pore size of 3.5 kDa and dialyzed against deionized water for several days until neutral pH values were achieved. The dialyzed solution of the K,Na-PHI nanoparticles was concentrated by evaporation at 60 °C to a concentration of 5.0 g L^{-1}, which was determined gravimetrically.

FTO substrate. The FTO substrates (Pilkington TEC 15, XOP Glass, Castellón Spain) were cleaned by sonication in acetone for 30 min followed by washing with deionized water. Then, the FTO substrates were boiled for 10 min in 0.1 M NaOH before washing again with deionized water and drying.

Preparation of the K,Na-PHI electrodes. The K,Na-PHI electrodes were fabricated following the procedure reported by Adler et al.[2] Briefly, 12.5 mL of the K,Na-PHI solution (4.5 g L^{-1}, pH ~8.5) was gelled by adding 32.5 mL of ethanol and centrifuged for 10 min at 8000 r.p.m. The formed gel was washed three times with ethanol before liquefication by vortexing. Afterward, 200 µL of the liquefied gel was smeared on two FTO glasses by the doctor blade technique using a scotch-tape as a frame and spacer. The fabricated electrodes were dried in air at room temperature and calcined at 450 °C under N_2 flow in a tube furnace with a heating rate of 5 °C min^{-1}.

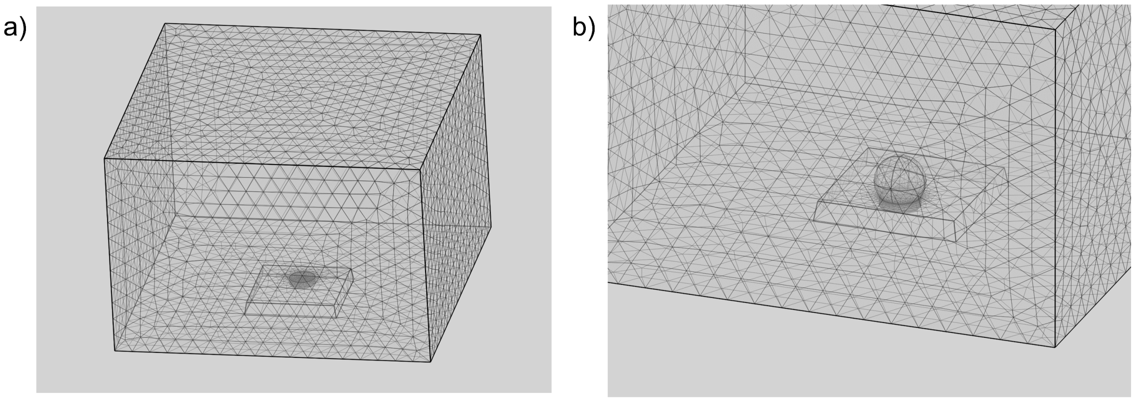

Figure S1. Boundary mesh for simulating the diffusion towards a) recessed disk electrode (diam. 4 μm) and b) colloidal probe (diam. 5 μm); simulations were performed with the COMSOL Multiphysics software.

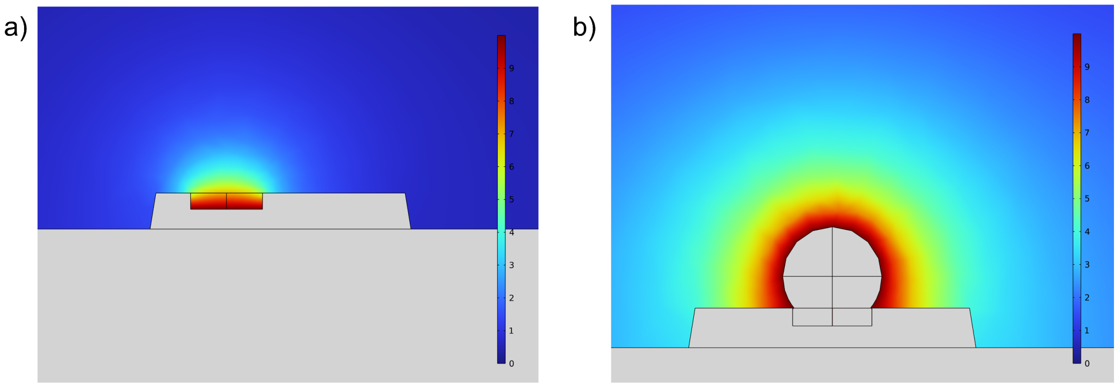

Figure S2. Concentration profile (scale bar in mM) of the diffusion-controlled mass transport of $[Ru(NH_3)_6]Cl_3$ at a) recessed disk electrode (diam. 4 μm), and b) colloidal probe (diam. 5 μm) obtained via the *Infinite Element* domain feature of the COMSOL Multiphysics software.

RESULTS AND DISCUSSION

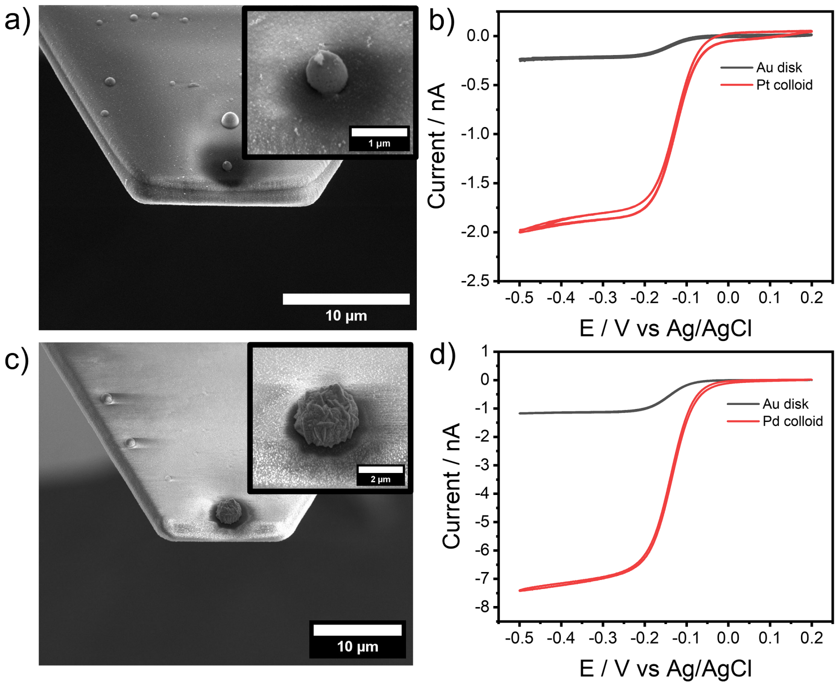

Figure S3. a) SEM images of the hemispherical Pt-B probe; inset zoomed view. Electrochemical deposition of Pt onto a recessed Au disk sub-microelectrode (diam. 0.5 μm) was carried out in 2.0 mM K_2PtCl_6 in 0.5 M H_2SO_4. 80 potential pulse cycles were applied (–0.06 V vs Ag/AgCl for 0.5 s and 0 V vs Ag/AgCl for 0.5 s). b) CVs before (grey) and after (red) electrodeposition of Pt-B recorded in 10 mM $[Ru(NH_3)_6]Cl_3$/0.1 M KCl, scan rate: 0.1 V s^{-1} (vs Ag/AgCl). c) SEM image of a Palladium (Pd)-modified probe (diam. 2.5 μm). Electrochemical deposition of Pd onto the recessed Au disk microelectrode (diam. 1 μm) was carried out in 20 mM K_2PdCl_6/0.5 M H_2SO_4. 75 potential pulse cycles were applied (0.35 V vs Ag/AgCl for 0.5 s and 0.6 V vs Ag/AgCl for 0.5 s). d) CVs before (grey) and after (red) electrodeposition of Pd recorded in 10 mM $[Ru(NH_3)_6]Cl_3$/0.1 M KCl, scan rate: 0.1 V s^{-1} (vs Ag/AgCl).

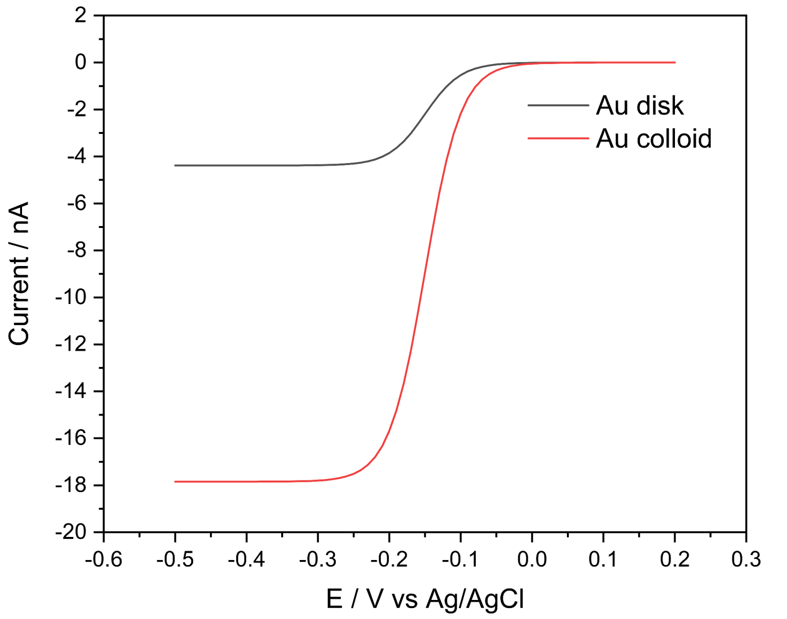

Figure S4. Simulated linear sweep voltammograms for the reduction of $[Ru(NH_3)_6]Cl_3$ based on diffusional mass transport towards a recessed disk microelectrode (diam. 4 μm, grey) and a colloidal probe (diam. 5 μm, red).

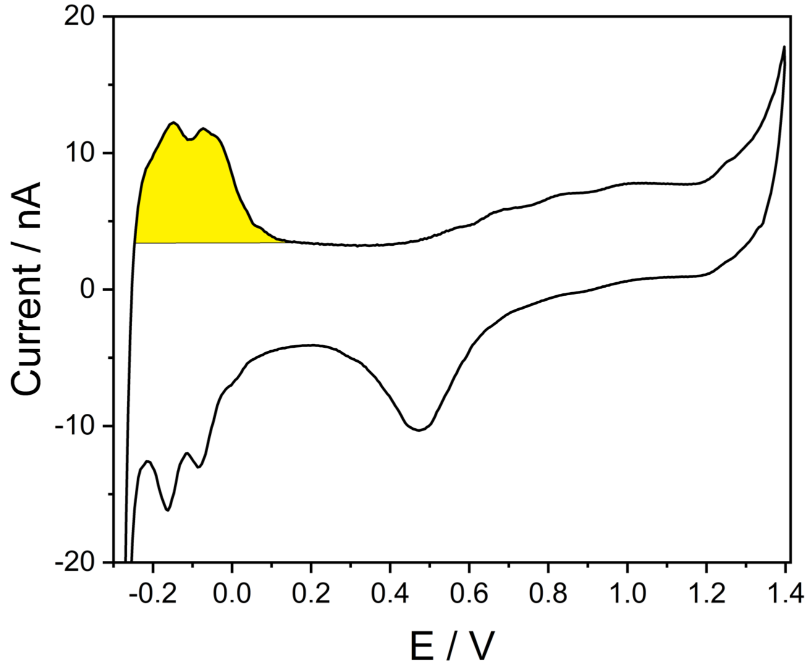

Figure S5. CV recorded at a hemispherical Pt-B probe in 0.5 M H_2SO_4, scan rate: 200 mV/s (vs Hg/Hg_2SO_4).

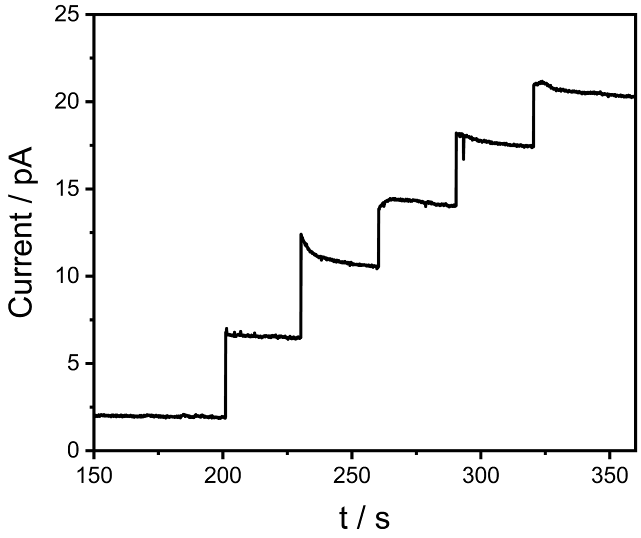

Figure S6. Amperometric *i*-*t*-curve for an exemplary H_2O_2 calibration recorded in PBS at pH 7.4 (applied potential at the hemispherical Pt-B probe: 0.35 V vs Ag/AgCl). Aliquots of 20 μL H_2O_2 stock solution were added (final concentration per addition: 2.5 μM).

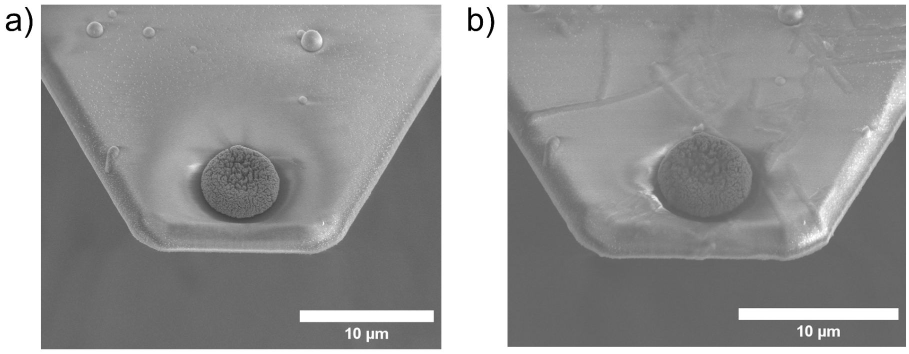

Figure S7. SE images of the hemispherical Pt-B probe prior a) and after b) imaging an Au substrate (30 × 30 μm) in contact mode.

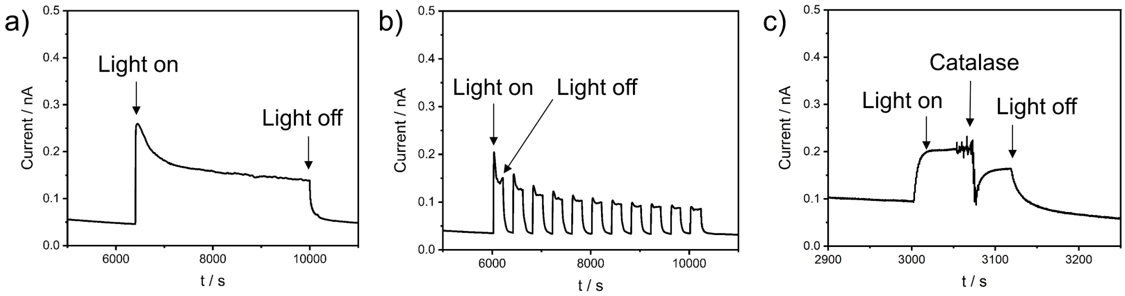

Figure S8. a) Amperometric (*i-t*) curve during illumination at λ = 365 nm in PBS containing 20% EtOH recorded at a Pt-B modified microelectrode (diam.: 25 μm) at a distance of 50 μm to the K,Na-PHI surface; applied potential 0.35 V vs Ag/AgCl. b) Amperometric response under chopped illumination (illumination step 200 sec and dark condition 200 sec). c) Control experiment adding 50 μL catalase (1 mg mL^{-1} in PBS) to the reaction solution (total volume: 4 mL).

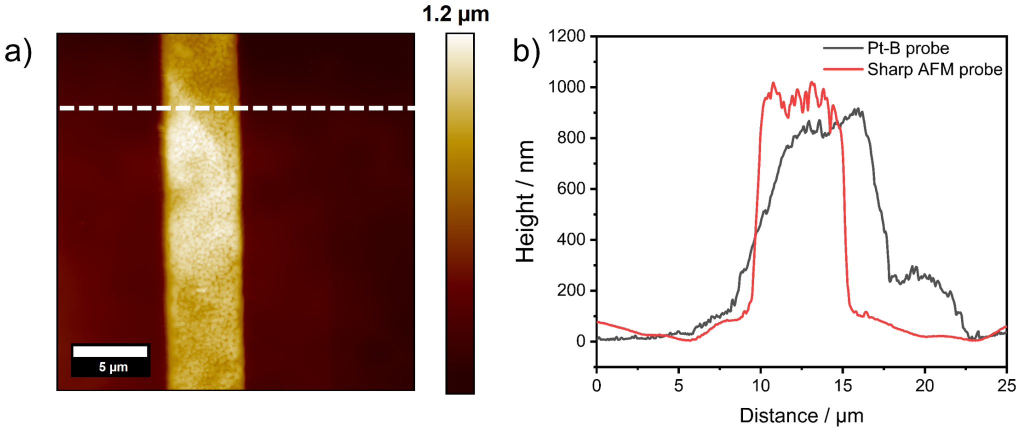

Figure S9. a) AFM topography of the K,Na-PHI ridge recorded with a commercial SiN AFM probe (nominal tip radius of 15 nm) and b) overlaid height profile with the hemispherical Pt-B probe showing the achievable horizontal resolution of the AFM probes.

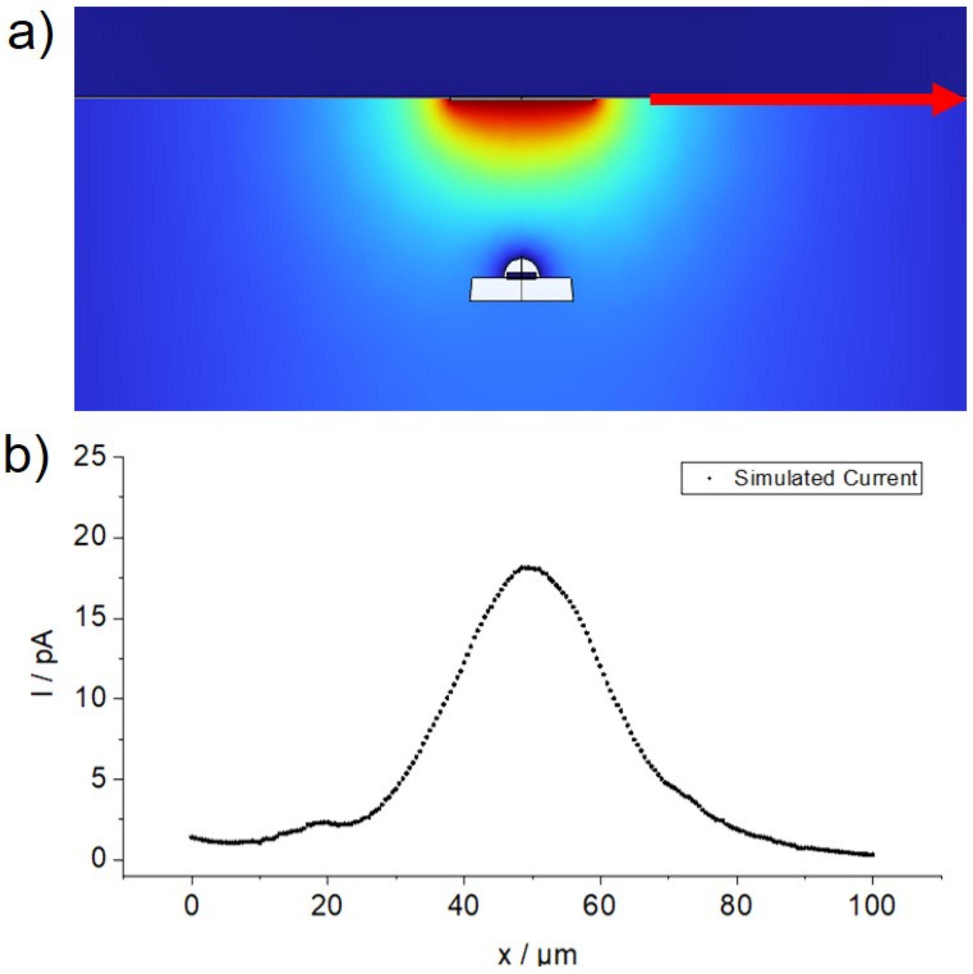

Figure S10. a) COMSOL simulation of current profile. The AFM-SECM probe was kept at a fixed position and the UME was moved in a distance of 20 µm across the probe. b) Simulated current profile at a distance of 20 µm from the UME.

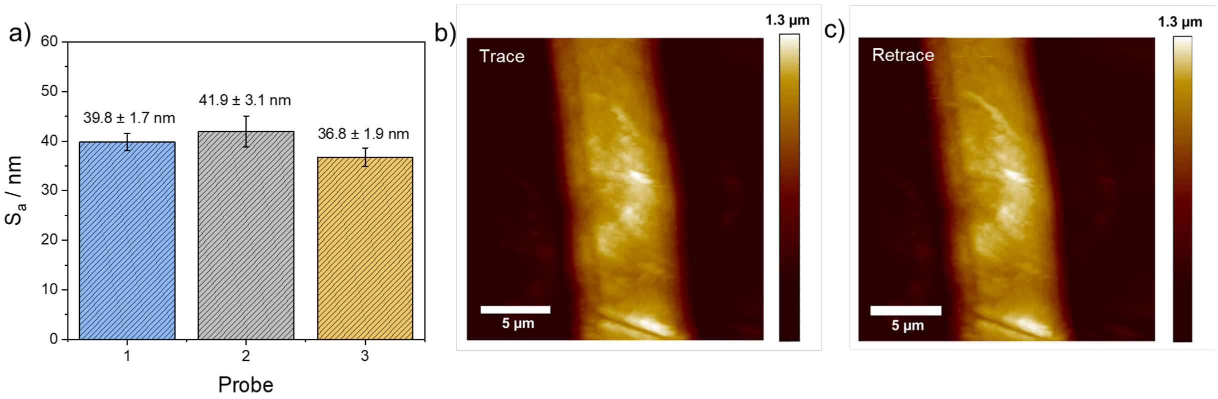

Figure S11. a) Bar diagram of surface roughness (S_a) of the K,Na-PHI ridge acquired with three individual hemispherical Pt-B probes (error bars reflect three individual spots) and b) trace and c) retrace AFM topography image of the K, Na-PHI ridge.

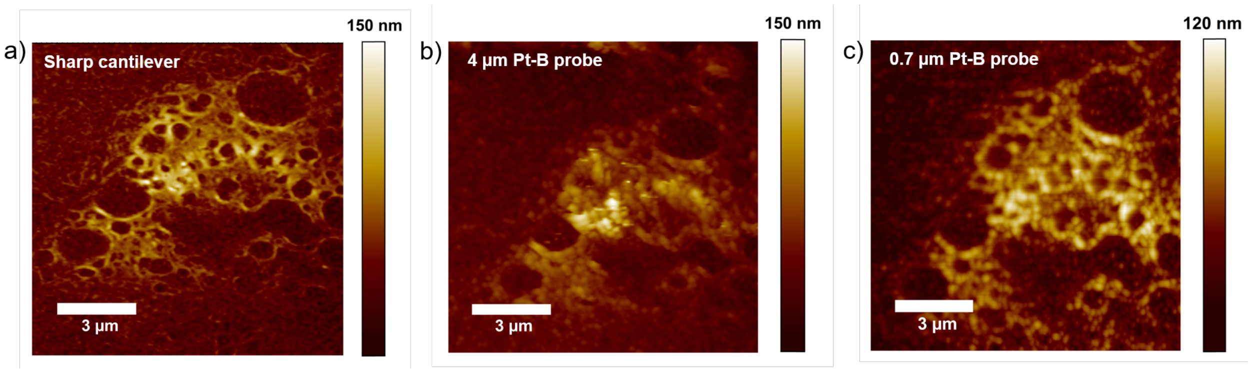

Figure S12. a) AFM topography of one K,Na-PHI particle recorded with a commercial SiN AFM probe, b) recorded with the hemispherical Pt-B probe (5.1 µm in diam.) and c) recorded with the 0.7 nm diam. Pt-B probe (shown in Figure S3a).

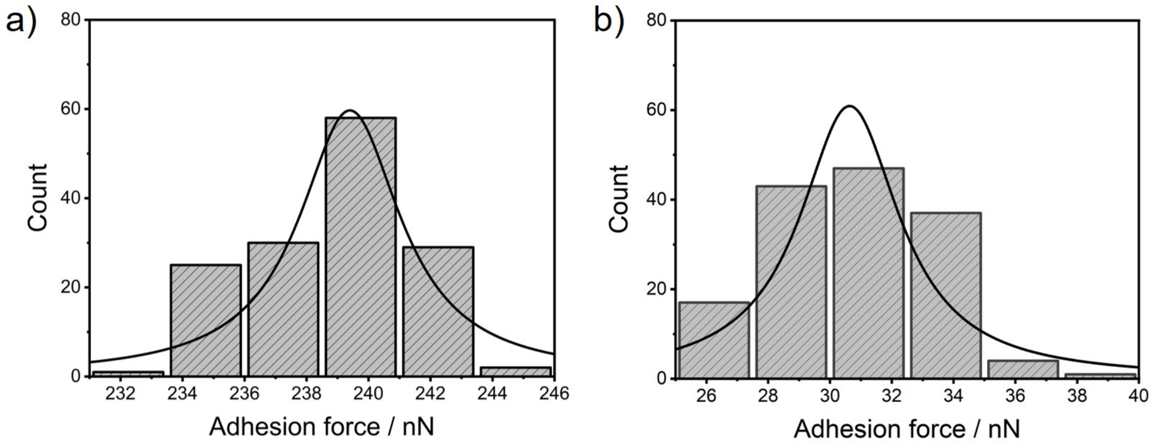

Figure S13. Histograms of the adhesion forces recorded in air; a) measurements on K,Na-PHI, and b) measurements on FTO (n = 150).

The bin widths were determined according to the following equation: Bin width = (Max value-Min value)/Number of bins. The number of bins were calculated by the square root of the total number of data points.

Supplementary references

(1) Krivtsov, I.; Mitoraj, D.; Adler, C.; Ilkaeva, M.; Sardo, M.; Mafra, L.; Neumann, C.; Turchanin, A.; Li, C.; Dietzek, B.; Leiter, R.; Biskupek, J.; Kaiser, U.; Im, C.; Kirchhoff, B.; Jacob, T.; Beranek, R. Water-Soluble Polymeric Carbon Nitride Colloidal Nanoparticles for Highly Selective Quasi-Homogeneous Photocatalysis. *Angew. Chemie Int. Ed.* **2020**, *59*, 487–495. https://doi.org/10.1002/anie.201913331.

(2) Adler, C.; Krivtsov, I.; Mitoraj, D.; Santos-Gómez, L.; García-Granda, S.; Neumann, C.; Kund, J.; Kranz, C.; Mizaikoff, B.; Turchanin, A.; Beranek, R. Sol−Gel Processing of Water-Soluble Carbon Nitride Enables High-Performance Photoanodes. *ChemSusChem* **2021**, *14*, 2170–2179. https://doi.org/10.1002/cssc.202100313.
